# Antitumor properties of Salvianolic acid B against triple-negative and hormone receptor-positive breast cancer cells via ceramide-mediated apoptosis

**DOI:** 10.18632/oncotarget.26348

**Published:** 2018-11-20

**Authors:** Wei Sha, Yanfei Zhou, Zhi-Qiang Ling, Guiqin Xie, Xiaowu Pang, Paul Wang, Xinbin Gu

**Affiliations:** ^1^ Departments of Oral Pathology, College of Dentistry, Howard University, Washington, D.C., USA; ^2^ TenGen Biomedical Co., Bethesda, Maryland, USA; ^3^ Zhejiang Cancer Hospital, Zhejiang Cancer Research Institute, Hangzhou, Zhejiang, China; ^4^ Department of Radiology, College of Medicine, Howard University, Washington, D.C., USA,; ^5^ Cancer Center, Howard University, Washington, D.C., USA; ^6^ College of Science and Engineering, Fu Jen Catholic University, Taipei, Taiwan

**Keywords:** salvianolic acid B, triple negative breast cancer, apoptosis, ceramides, glucosylceramide synthase

## Abstract

Triple-negative breast cancer (TNBC) is an aggressive subtype of breast cancer with limited treatment options. It is urgent to develop new therapeutics against this disease. Salvinolic acid B (Sal-B) is a leading bioactive component of Salvia *miltiorrhiza* Bunge, a well-known Chinese medicine for treating various diseases without appreciable adverse effects. To understand the antitumor properties of Sal-B against TNBC, we analyzed its effects on the cell viability, cell cycle and apoptosis of triple-negative MDA-MB-231 cells with the hormone receptor-positive MCF-7 cells as the control. The *in vitro* analysis showed that Sal-B could significantly reduce the cell viability and suppress the proliferation of both MDA-MB-231 and MCF-7 cells with decreased cyclin B1 expression, but with no noticeable cell cycle phase change. In mouse models, Sal-B markedly inhibited the growth, decreased the PCNA expression, and increased the cell apoptosis of MDA-MB-231 tumor xenografts. To understand the antitumor mechanisms, we analyzed the expression levels of ceramides, and anti-apoptotic (Bcl-xL and survivin) and pro-apoptotic (caspase-3 and caspase-8) proteins. We found that Sal-B enhanced the ceramide accumulation and inhibited the anti-apoptotic protein expression. Interestingly, the ceramide accumulation was accompanied by decreased expression of glucosylceramide and GM3 synthases, two key enzymes regulating ceramide metabolism. These findings indicate that Sal-B exerts its antitumor effects at least partially by inducing the ceramide accumulation and ceramide-mediated apoptosis via inhibiting the expression of glucosylceramide and GM3 synthases, which was independent of estrogen receptor α. Sal-B appears to be a promising therapeutic agent against TNBC.

## INTRODUCTION

Breast cancer is the most common cancer in women and the second highest lethal form of cancer in the United States [1]. Studies have shown that the incidence of breast cancer has been gradually decreasing in the last two decades, however women in the United States still have a one in eight chance of developing breast cancer in their lifetime [2]. Critically, about one third of breast cancer is triple-negative (TNBC), presenting the lack of expression of estrogen receptor α (ER-α), progesterone receptor, and human epidermal growth factor receptor-2 (HER2) [3]. Due to the poor response to anti-hormonal treatment and chemotherapeutics, TNBC is considered an aggressive form of breast cancer [3]. The commonly used therapeutic agents for breast cancer including doxorubicin have little success in treating patients with TNBC [4]. In addition, the cardiotoxicity risk of doxorubicin also limits its clinical applications [5, 6].

The sphingolipids, such as ceramides, play an important biological role in the regulation of proliferation, differentiation, and apoptosis of cancer cells [7–9]. Intracellular ceramide accumulation has been shown to enhance the apoptosis of cancer cells [10–12]. Development of drug resistance of cancer cells is also associated with an increase in ceramide glycosylation [13–15]. Glucosylceramide synthase (GCS) is a key enzyme in the metabolism of ceramide glycosylation, which converts ceramide to glucosylceramide. Early inhibition of GCS has potential to prevent the drug resistance [13]. In addition, the TNF-α-induced apoptosis pathway is also involved in the process of increasing intracellular ceramides [16].

Salvianolic acid B (Sal-B) is a natural compound extracted from *Salvia Miltorrhiza* Bunge, a well-known Chinese herbal medicine for preventing and treating vascular diseases. Sal-B is also a quality control ingredient and an active marker for *S. Miltorrhiza* Bunge products by the National Pharmacopoeia Council of China [17–19]. In the previous studies, we have found that Sal-B has a suppressing effect against head and neck squamous carcinoma cells [19]. This anticancer effects of Sal-B have been shown to involve in various mechanisms [20–26]. A recent study has revealed that the effect of Sal-B on human glioblastoma U87 cells is through p38 activation-mediated reactive oxygen species generation [22]. In the present study, we analyzed the antitumor properties of Sal-B against triple-negative MDA-MB-231 cells using the hormone receptor-positive MCF-7 cells as the control. The results showed that Sal-B had a high potency against TNBC, which was mediated by inhibiting the tumor cell growth and enhancing the ceramide-mediated apoptosis through GCS-catalyzed ceramide glycosylation.

## RESULTS

### Sal-B inhibited the growth of both triple-negative and hormone receptor-positive breast cancer cells *in vitro*

**Figure 1:**
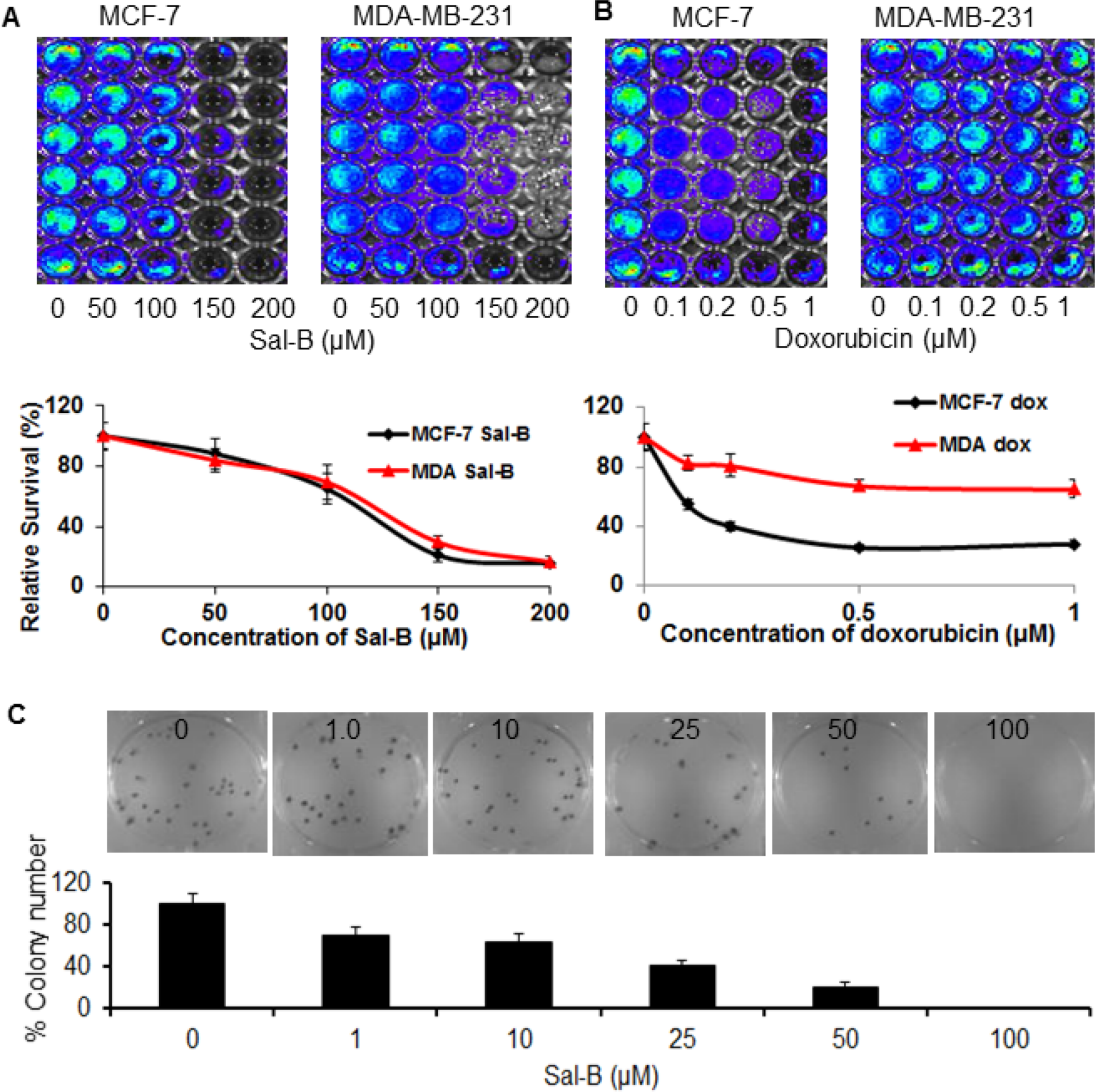
Inhibitory effects of Sal-B on the cell viability and colony formation of both triple-negative MDA-MB-231 and hormone-receptor positive MCF-7 breast cancer cells The cell viability was analyzed by luciferase-based bioluminescent imaging after exposure to Sal-B (50 µM, 100 µM, 150 µM and 200 µM) (**A**) and doxorubicin (0.1 µM, 0.2 µM, 0.5 µM and 1 µM) (**B**) for 24 h, respectively. The colony formation of MDA-MB-231 cells was determined with colony formation assay after exposure to various doses of Sal-B (1 µM, 10 µM, 25 µM, 50 µM and 100 µM) for 24 h and colonies were allowed to grow for 10 days (**C**). A colony containing more than 50 cells was considered to represent a viable clonogenic cell. The results represent the mean ± SD. There were significant differences for the cell viability and colony formation capability between untreated and Sal-B treated cells (*P* < 0.05). No significant difference was observed for the effect of Sal-B on the cell viability between MDA-MB-231 and MCF-7 cells (*P* > 0.05), different from that of doxorubicin (*P* < 0.05).

We used the TNBC MDA-MB-231 cell line and hormone receptor-positive MCF-7 cell line to test the effectiveness of Sal-B and doxorubicin on the cancer cell viability (Figure 1A and 1B). Both cell lines have been stably transfected and expressed the firefly luciferase gene. The bioluminescent signal intensity reflects the cell metabolic activity, being highly correlated with the cell viability. Therefore we evaluated the cell viability using the sensitive bioluminescent optical imaging. The cells were treated with Sal-B (50, 100, 150, and 200 µM) and doxorubicin (0.1, 0.2, 0.5, and 1 µM), respectively, for 24 hours. We observed a similar inhibitory effect of Sal-B on the cell viability of both MDA-MB-231 and MCF-7 cells in a dose-dependent manner. Compared with untreated cells, MDA-MB-231 and MCF-7 cells decreased their cell viabilities to 69% and 65%, respectively, when treated with Sal-B at 100 µM for 24 hours (Figure 1A). The half maximal inhibitory concentrations (IC_50_) were 125 µM for MDA-MB-231 cells and 120 µM for MCF-7 cells. There was no significant difference for the inhibitory effect of Sal-B between the two cell lines. Differentially, doxorubicin was more effective on the inhibition of MCF-7 than MDA-MB-231 cell growth (Figure 1B). At 0.5 µM concentration, doxorubicin reduced the viability of MCF-7 cells by approximately 67%, whereas there was only 25% reduction for the MDA-MB-231 cells (*P* < 0.05). No further reduction of the cell viability was observed in both cell lines treated with 1 µM doxorubicin.

### Sal-B suppressed the colony formation of breast cancer cells

The effect of Sal-B on the cancer cells was further tested with colony formation assay (Figure 1C). The colony contained more than 50 cells was considered to represent a viable clonogenic cell after 10 days following exposure for 24 hours to Sal-B at varied concentrations (1, 10, 20, 50, and 100 µM). As shown in Figure 1C, the colony formation capability of the cells treated at the concentration of 1 µM was approximately 69% of that of the control cells, and a complete inhibition of the cell colony formation capability was observed at 100 µM.

**Figure 2:**
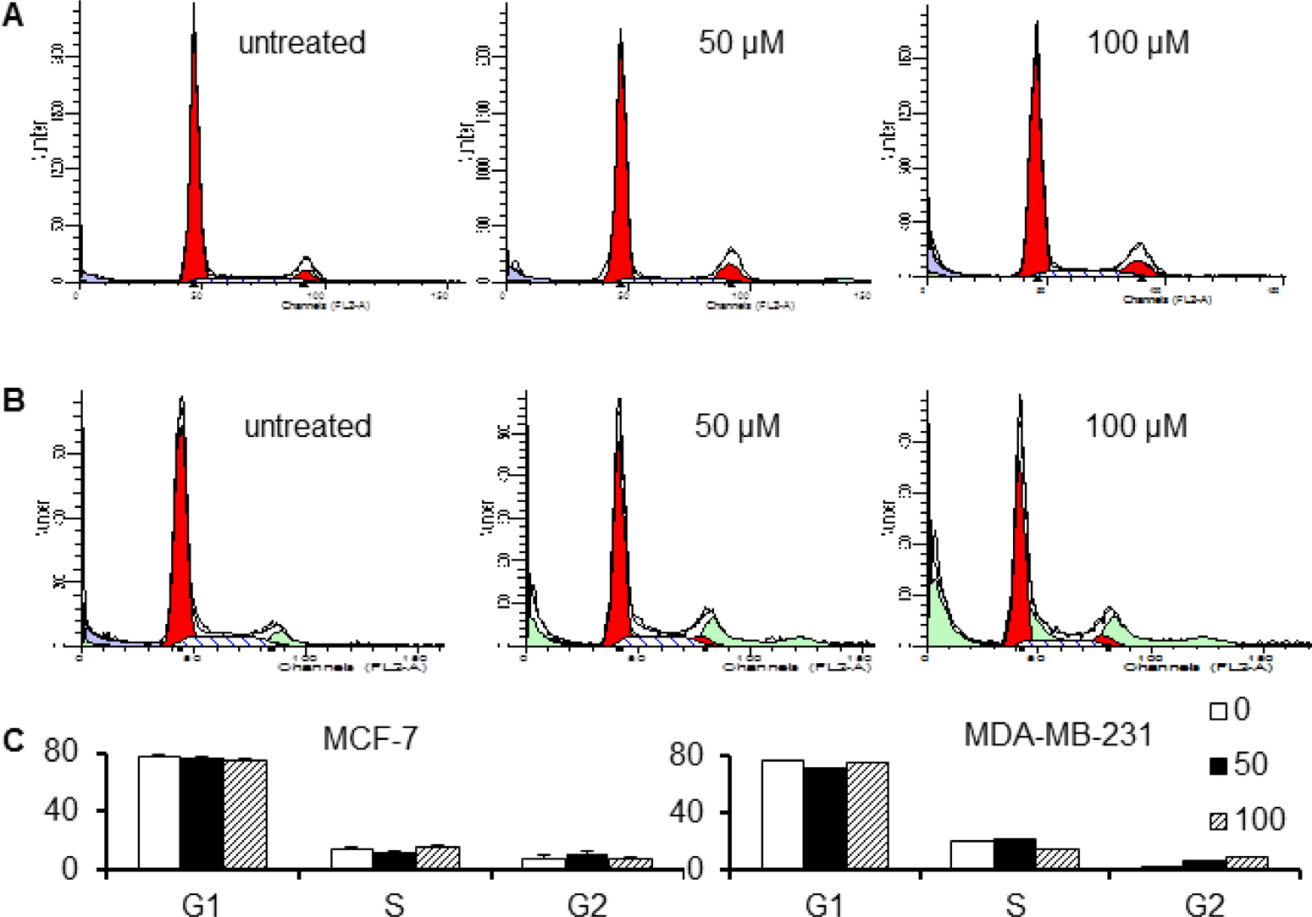

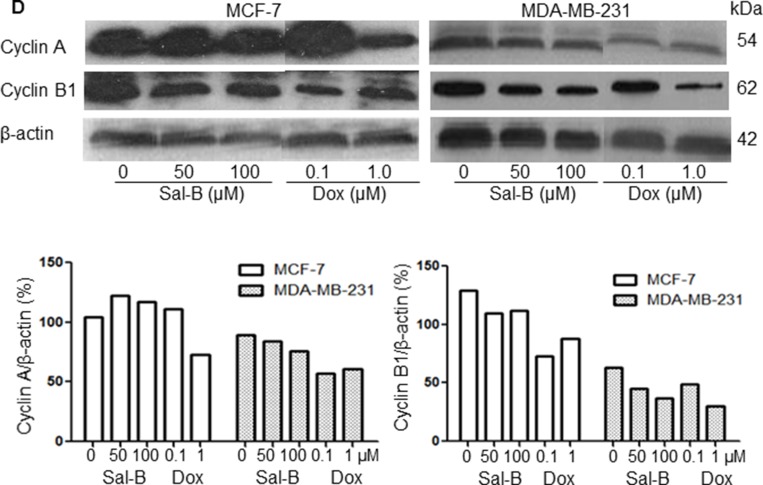
Effects of Sal-B on cell cycle and cell cycle related protein expression in MCF-7 and MDA-MB-231 cells (**A**, **B**) show the DNA content profiles of MCF-7 and MDA-MB-231 cells, respectively, by flow cytometry. The percentages of cells in the different phases of cell cycle are summarized as mean ± SD (**C**). The upper panel of (**D**) shows the Western blot analysis for the expression of cell cycle related protein cyclin A and cyclin B1. The expression of cyclin A and cyclin B1 was normalized by the expression of the internal control β-actin (lower panel). The cells were treated with 50 µM and 100 µM Sal-B, respectively, for 24 h. Doxorubicin (0.1 µM and 1 µM) was used as a control. No significant change was observed in the cell cycle phases of either MDA-MB-231 or MCF-7 cells after Sal-B treatment. For the cyclin A and cyclin B1 expression, down-regulation of cyclin B1 but not cyclin A expression was found in both Sal-B treated MDA-MB-231 and MCF-7 cells. Treatment with 1 µM doxorubicin resulted in reduction of both cyclin A and cyclin B1 expression in both cell lines.

To understand the effects of Sal-B on the cell cycle of cancer cells, we analyzed the cell cycle profile using flow cytometry. There were no significant changes in the cell cycle phases for either MDA-MB-231 or MCF-7 cells after exposure for 24 hours to Sal-B at concentrations of 50 µM (Figure 2A and 2C) and 100 µM (Figure 2B and 2C). When checking the expression of two cell cycle-related proteins, cyclin A and cyclin B1, we observed that Sal-B was able to down-regulate the cyclin B1 expression in both MDA-MB-231 and MCF-7 cells, but had no significant effect on cyclin A protein expression (Figure 2D). Treatment with 1 µM doxorubicin resulted in a significant reduction of both cyclin A and cyclin B1 expression in both cell lines.

### Inhibitory effect of Sal-B on the growth of TNBC MDA-MB-231 tumor xenografts

**Figure 3:**
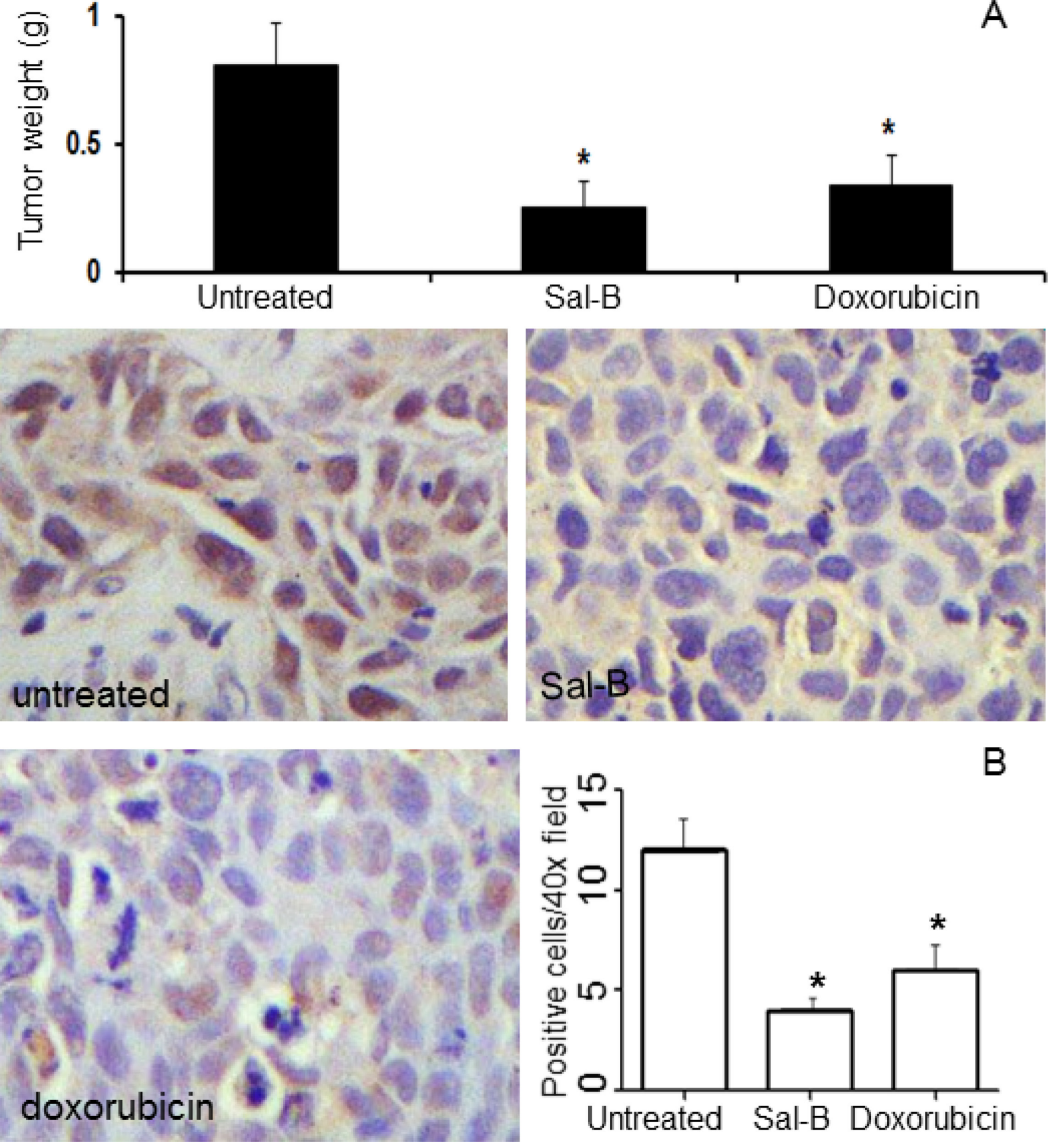
Inhibitory effects of Sal-B on the growth of triple-negative MDA-MB-231 tumor xenografts in nude mice The mice were treated with either Sal-B (80 mg/kg three times per week) or doxorubicin (4 mg/kg every 4 weeks) and all mice were sacrificed on day 37. (**A**) shows the tumor weights on day 37. The PCNA levels were higher in untreated tumors than in Sal-B treated and doxorubicin treated tumors, as determined by immunohistochemical analysis (**B**). The results represent the mean ± SD and ^*^P < 0.05 means significant difference with respect to control mice.

The above findings showed that Sal-B could inhibit the proliferation of MDA-MB-231 cells *in vitro*. We thus tested the effects of Sal-B on the growth of MDA-MB-231 tumor xenografts in animals. The treatment was initiated 10 days after MDA-MB-231 cells were transplanted into the nude mice, and at that time, the tumor mass in each mouse was about 5 mm in diameter. Our previous studies have shown that treatment with Sal-B at 80 mg/kg three times per week leads to no obvious side effects, but a higher dosage has resulted in the weight loss in some mice. Also based on publications regarding doxorubicin, the agents were administered by intraperitoneal injection with Sal-B at 80 mg/kg three times per week, and doxorubicin at 4 mg/kg every four weeks for a total of 37 days. As shown in Figure 3A, the average tumor weights of Sal-B treated group (0.26 ± 0.06 g) and doxorubicin treated group (0.34 ± 0.09 g) were both significantly smaller than that of untreated control group (0.81 ± 0.12 g) (both *P* < 0.05). During the experimental period, body weights of mice were measured every week and there were no significant differences between untreated mice and treated mice (data not shown). In parallel with the decreased tumor growth, a lower expression level of proliferating cell nuclear antigen (PCNA) was observed in Sal-B treated and doxorubicin treated tumor xenografts than in untreated MDA-MB-231 tumor xenografts (Figure 3B).

### Effects of Sal-B on the expression of apoptosis-related proteins and apoptosis in MDA-MB-231 and MCF-7 cancer cells

**Figure 4:**
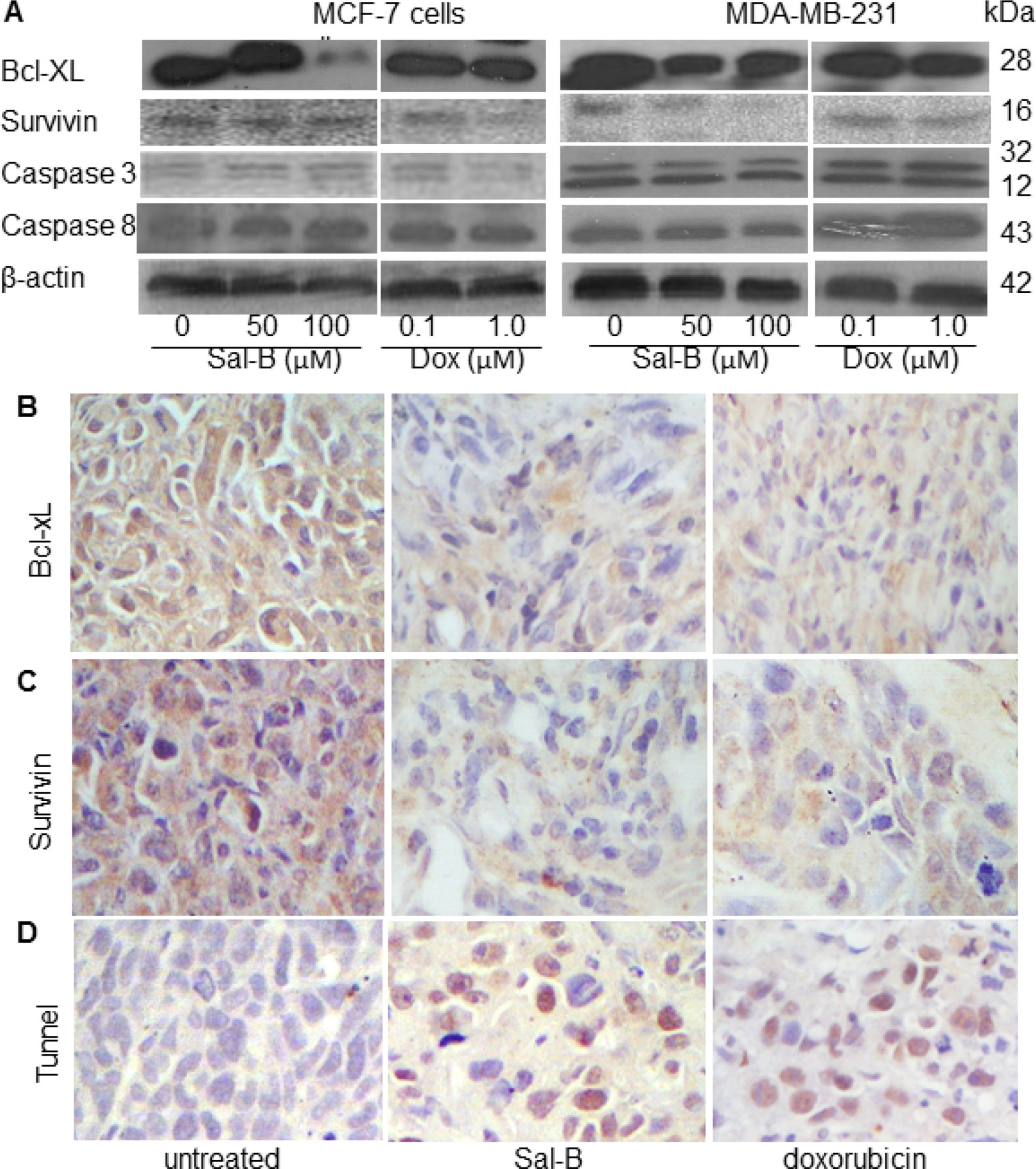
Effects of Sal-B on the apoptosis in both MCF-7 and MDA-MB-231 cancer cells and tumor xenografts Western blot analysis was used to determine the expression of apoptosis-related proteins Bcl-xL, survivin, caspase-3 and caspase-8 (**A**). β-actin was used as an internal control for Western blotting. The cells were treated with Sal-B (50 µM and 100 µM) and doxorubicin (0.1 µM and 1 µM), respectively, for 24 h. Both Sal-B and doxorubicin downregulated both Bcl-xL and survivin protein expression, but not the protein levels of caspase-3 and caspase-8 in the MDA-MB-231 and MCF-7 cell lines. Immunohistochemical analysis was used to detect the expressions of Bcl-xL (**B**) and survivin (**C**) in MDA-MB-231 tumor xenografts, showing decreased expression of Bcl-xL and survivin in the Sal-B and doxorubicin treated tumors than in the untreated tumors (*P* < 0.05). Apoptotic cells in MDA-MB-231 tumor xenografts were analyzed by a TUNEL assay, showing an increased apoptotic cells in the Sal-B and doxorubicin treated xenografts than in the untreated tumors (*P* < 0.05) (**D**).

The effect of Sal-B on the apoptosis of MDA-MB-231 and MCF-7 cells was analyzed *in vitro* and in tumor xenografts with different methods. We first investigated the levels of apoptosis-related proteins *in vitro*, including two anti-apoptotic (Bcl-xL and survivin) and two pro-apoptotic (caspase-3 and caspase-8) proteins using Western blot analysis (Figure 4A). Sal-B treatment downregulated both Bcl-xL and survivin protein expression, but not the protein levels of caspase-3 and caspase-8 in the MDA-MB-231 and MCF-7 cells. Similar results to Sal-B were observed for doxorubicin. The expression of Bcl-xL and survivin in MDA-MB-231 tumor xenografts was further analyzed by immunohistochemistry. The expression of Bcl-xL decreased significantly in the Sal-B treated tumors (1.3 ± 0.4) than in untreated tumors (11.8 ± 3.0) (*P* < 0.05) (Figure 4B). Survivin level showed similar changes, decreasing in Sal-B and doxorubicin treated groups compared to that in untreated group (Figure 4C). The immunohistochemical results were consistent with those from Western blotting.

We then investigated the apoptotic cells in the tumors using TUNEL assay. As the results, the highest number of apoptotic cells was found in the Sal-B treated xenografts (12.2 ± 3.2), followed by doxorubicin treated xenografts (9.0 ± 2.1), significantly different from that in the untreated tumors (3.1 ± 1.8) (both *P* < 0.05) (Figure 4D).

### Sal-B increased ceramide accumulation and inhibited GCS and GM3 synthase expression

**Figure 5:**
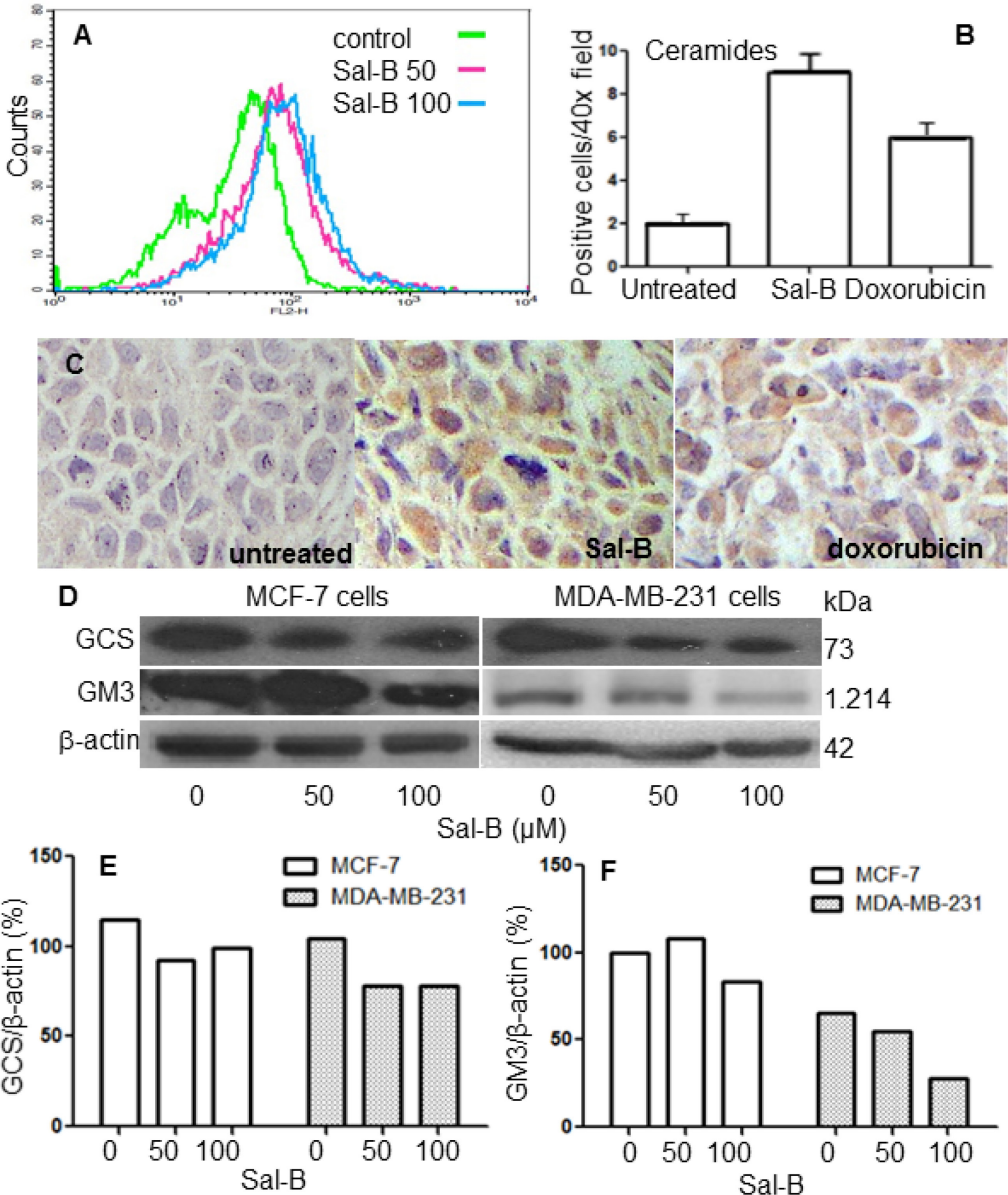
Sal-B enhances ceramide accumulation and decreases the expression of GCS and GM3 enzymes MDA-MB-231 cells were treated with 50 µM and 100 µM of Sal-B, respectively, for 24 h and ceramide levels were then analyzed by flow cytometry (**A**). Immunohistochemistry was used to evaluate the expression of ceramides in MDA-MB-231 tumor xenografts (**C**) and the ceramide positive cells were counted (**B**). The expression of GCS and GM3 synthase in MDA-MB-231 and MCF-7 cells treated by various concentrations of Sal-B was determined with Western blotting (**D**). The amount of protein expression was normalized by the expression of the internal control β-actin (**E** and **F**).

Ceramide level was analyzed with flow cytometry through comparison between Sal-B treated and untreated MDA-MB-231 cells. We observed that 50 µM of Sal-B was sufficient to enhance the ceramide level (Figure 5A). In line with this result, immunohistochemical analysis revealed that ceramide level significantly increased in both Sal-B treated (9.6 ± 2.4) and doxorubicin treated (6.2 ± 1.3) MDA-MB-231 tumor xenografts, compared to that in untreated control (2.3 ± 0.7) (both *P* < 0.05) (Figure 5B and 5C). To understand the mechanism underlying increased ceramide accumulation, we measured the expression levels of GCS and GM3 synthases using Western blot analysis. As shown in Figure 5D and 5E, a low dose (50 µM) of Sal-B was sufficient to inhibit GCS protein level in both MCF-7 and MDA-MB-231 cells. There was no further inhibitory effect on GCS expression when treated with a higher dose (100 µM), compared with the low dose (50 µM). The expression of GM3 synthase was also significantly diminished in the Sal-B treated MDA-MB-231 cells at both 50 µM and 100 µM, and in the MCF-7 cells at 100 µM (Figure 5D and 5F).

### No effects of Sal-B on the ER-α and p-ERK expression in breast cancer cells

**Figure 6:**
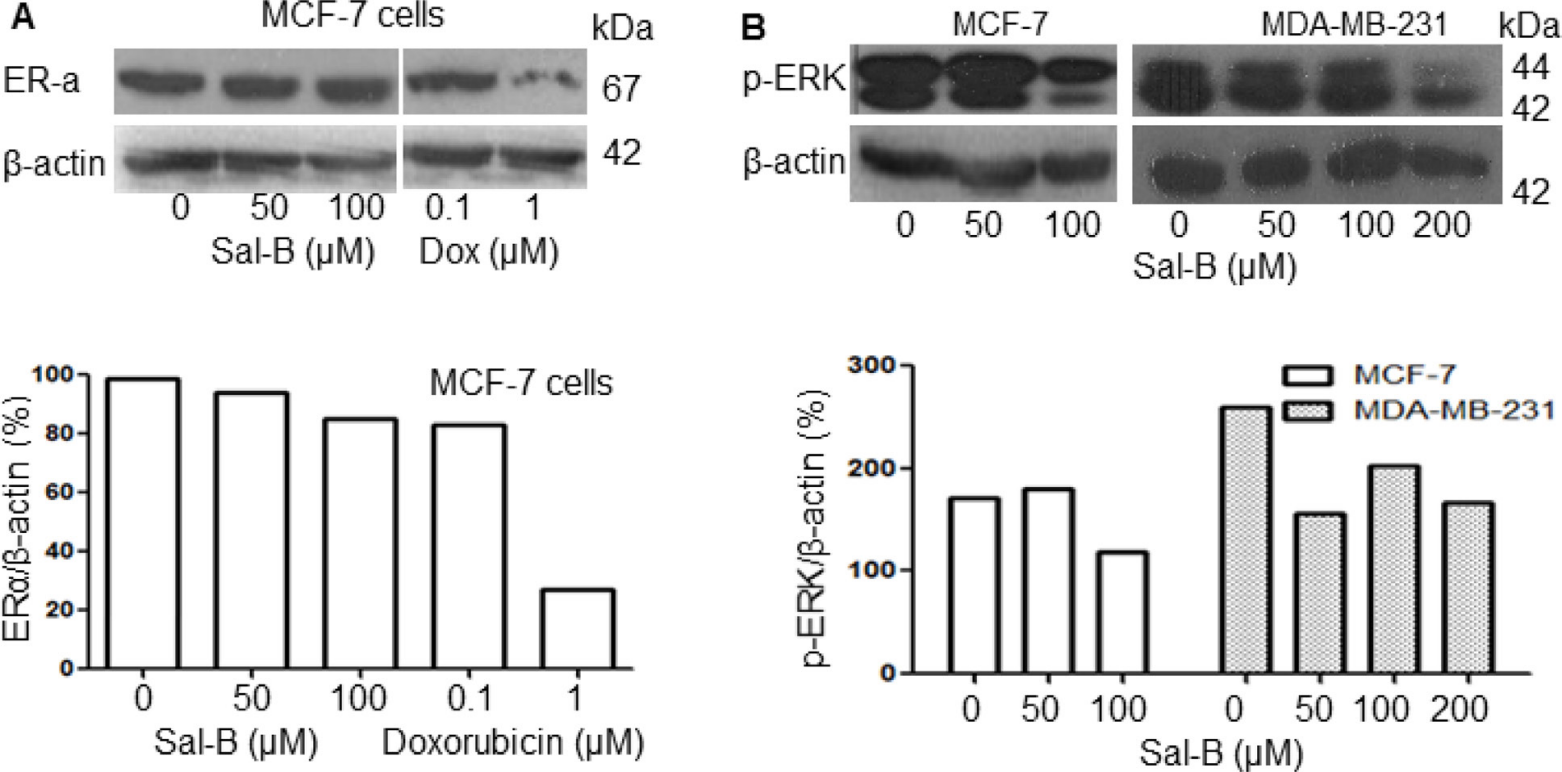
Effects of Sal-B on the ER-α and p-ERK expression in breast cancer cells with Western blot analysis (**A**) shows the ER-α expression in MCF-7 cells treated with Sal-B and doxorubicin (upper panel) and (**B**) shows the expression of p-ERK in MCF-7 and MDA-MB-231 cells following exposure to different concentrations of Sal-B for 24 hours (upper panel). The amount of protein was normalized by that of the internal control β-actin (lower panel).

To understand whether Sal-B regulated the expression of ER-α, we investigated the ER-α expression in MCF-7 cells. The results showed that ER-α protein level was decreased by exposure to doxorubicin at the high dose (1 µM), but not to Sal-B (Figure 6A). Considering that phospho-p44/42 extracellular signal-regulated kinases (p-ERK) is a key enzyme in the MAPK signal pathway, we checked the expression of p-ERK and found that it was down-regulated by Sal-B in both MCF-7 and MDA-MB-231 cells (Figure 6B).

## DISCUSSION

TNBC is an aggressive subtype of breast cancer, which tends to grow and spread more quickly than other subtypes [27–30]. Available endocrine and HER2-directed therapies are ineffective against TNBC. Chemotherapy has been shown to be the most effective treatment for TNBC with the anthracycline drug, doxorubicin, being considered to be one of the most effective chemotherapeutic agents [31–35]. However, the clinical efficacy of doxorubicin is limited by the drug resistance development and severe side effects [36–39]. There is an urgent need to develop new therapeutics against TNBC.

Our previous studies have indicated that Sal-B is a unique natural anticancer agent that effectively inhibits the growth of head and neck cancer without significant side effects [19, 24]. In this study, we evaluated the antitumor property of Sal-B against TNBC MDA-MB-231 cells and compared it with that against hormone receptor-positive MCF-7 cells. We first found that Sal-B was able to reduce the cell viability of both MDA-MB-231 and MCF-7 cells *in vitro*; showing an IC_50_ of 125 µM for MDA-MB-231 cells and 120 µM for MCF-7 cells. No significant difference was observed for this effect of Sal-B between the two cell lines. Differently, the effect of doxorubicin (1 µM) on the cell viability was approximately two-fold higher for MCF-7 cells than for MDA-MB-231 cells. The inhibitory effect of Sal-B was further confirmed by colony formation assay. These results indicate that Sal-B has an inhibitory effect on TNBC cell growth *in vitro*.

We then tested the effect of Sal-B on the cell cycle progression of TNBC cells. We did not found a significant change in each phase of the cell cycle for both MDA-MB-231 and MCF-7 cells after exposure for 24 hours to Sal-B at concentration of either 50 µM or 100 µM, but Sal-B down-regulated the expression of cyclin B1. The results may be explained by that the cell cycle progression is controlled by a large set of molecules, although cyclin B1 is a key component in the control of cell cycle progression from G2 to M phase. Differently, doxorubicin effectively decreased the expression of both cyclin A and cyclin B1 in both cell lines. The expression of cell cycle related proteins has been found to be higher in doxorubicin-resistant breast cancer cells and doxorubicin could induce prostate cancer apoptosis by inhibiting cyclin B1 expression [39, 40].

The results obtained from *in vitro* studies were consistent with those from the animal model of TNBC. In the animals, Sal-B significantly inhibited the growth of MDA-MB-231 tumor xenografts. To understand the mechanisms underlying the antitumor effect of Sal-B, we investigated whether Sal-B could induce apoptosis of MDA-MB-231 tumor xenografts. As the results, a higher number of apoptotic cells was found in the Sal-B treated tumors than the control tumors, and the increased apoptosis was accompanied by ceramide accumulation in cancer cells *in vitro* as well as in tumor xenografts. These results suggest that ceramide-mediated cell apoptosis may play a critical role in the effect of Sal-B against TNBC. To further understand the ceramide metabolism, we analyzed the expression of GCS and GM3 synthases. GCS is a key enzyme to convert ceramide to glucosylceramide, catalyzing the first reaction of ceramide glycosylation in sphingolipid metabolism, while GM3 synthase catalyzes the initial step in the biosynthesis of most complex gangliosides from lactosylceramide. Both synthases closely regulate the ceramide metabolism. We found that Sal-B effectively down-regulated GCS and GM3 enzyme expression in cancer cells. Suppression of GCS and GM3 enzymes by Sal-B may lead to the accumulation of ceramides in cancer cells. Doxorubicin has also been shown to induce ceramide accumulation in cancer cells by other studies [41–43]. Both Sal-B and doxorubicin may induce TNBC cell apoptosis through the ceramide-mediated pathway.

**Figure 7:**
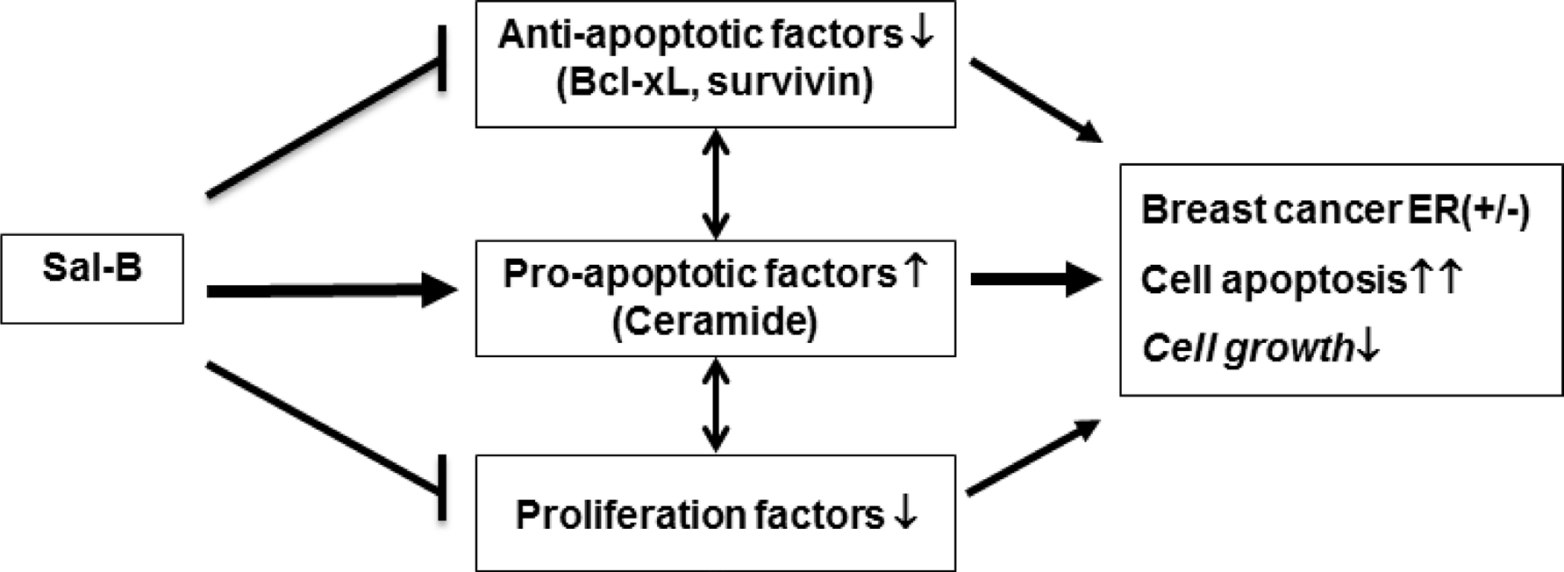
Proposed mechanisms of the antitumor effects of Sal-B on breast cancer cells Sal-B depresses cell growth and induces cell apoptosis in triple-negative breast cancer cells *via* (1) decreasing expression of anti-apoptotic factors (Bcl-XL and survivin); (2) enhancing expression of pro-apoptotic factors (i.e., ceramide); and (3) inhibiting proliferation factors. The antitumor activity of Sal-B suggests that Sal-B is highly promising to be a new therapeutic agent against triple-negative breast cancer.

Doxorubicin is known to induce apoptosis of breast cancer cells by regulating the expression of apoptosis-related proteins and some members of caspase-family enzymes [44]. Keeping these in mind, we studied whether Sal-B could regulate the expression of anti-apoptotic proteins (Bcl-xL and survivin) and caspase enzymes (caspase-3 and caspase-8), similar to doxorubicin. Our results showed that neither Sal-B nor doxorubicin could significantly change the caspase-3 and caspase-8 in TNBC cells, suggesting the less involvement of death-receptor (extrinsic) pathway. Interestingly, both Sal-B and doxorubicin inhibited Bcl-xL and survivin expression, indicating the involvement of the mitochondrial (intrinsic) pathway. Taken these together, we propose that the antitumor effect of Sal-B against TNBC is taken at least partially through a ceramide-centered mechanism (Figure 7).

The MAPK/ERK pathway is a chain of proteins in the cells, which communicates a signal from a receptor on the surface of cells to the DNA in the nucleus of the cells, critical for establishing solid tumor growth, especially for breast cancer. P-ERK is a critical member in MAPK signal pathway and also a marker of the activation of MAPK signal pathway in up-regulating cell proliferation. We observed that Sal-B significantly decreased the p-ERK expression in both cell lines, suggesting that Sal-B might inhibit cell proliferation of TNBC cells by regulating MAPK signal pathway. Ceramides may interact with the MAPK pathway through inhibiting the p-ERK expression, but the mechanism is still not clear. A recent study has shown that the three receptor tyrosine kinases c-KIT, VEGFR2 and PDGFRα are increased in TNBC [44].

In summary, our studies demonstrated that Sal-B could effectively inhibit the growth of both culture MDA-MB-231 cells and tumor xenografts through a ceramide-mediated pathway. Sal-B enhanced the cell apoptosis and decreased cell proliferation of TNBC by regulating the ceramide glycosylation enzymes. Given the fact that the currently available chemotherapeutic agents including doxorubicin are usually associated with severe side effects and less effective against TNBC, Sal-B provides promise as an effective anticancer agent with low toxicity, although studies are necessary to further elucidate the antitumor effects on other types of cancer and the underlying mechanisms. It is also interesting to investigate whether combination of Sal-B and other chemotherapeutic agents will provide an effective therapeutic strategy to improve the clinical outcome.

## MATERIALS AND METHODS

### Chemical reagents and antibodies

Sal-B was isolated and purified from *Salvia miltiorrhiza* Bunge as described previously [19]. Briefly, Sal-B was first extracted from *Salvia miltiorrhiza* Bunge powder with 70% ethanol in Soxhlet extractor. The extract was then passed through a D101 Macroporous resin and the magnesium salt of Sal-B was eluted with a six-fold column volume of 20∼40% ethanol solutions. The Sal-B magnesium salt-rich fraction in 40% ethanol was concentrated and converted into free Sal-B by adjusting to pH 3–4 with hydrochloric acid. The free Sal-B was dried and dissolved in water and purified with a polyamide chromatographic column. The highly purified Sal-B (>95%) was analyzed with high-pressure liquid chromatography. Before use, the purified Sal-B was dissolved in molecular grade water.

Doxorubicin was extracted from commercially available Adriamycin solution (Bedford Lab, Bedford, OH). The monoclonal or polyclonal antibodies against ceramides, survivin, Bcl-xL, caspase-3, caspase-8, cyclin A, cyclin B1, and PCNA were purchased from Sigma (St. Louis, MO). The anti-ceramide monoclonal antibody (clone: MID 15B4) recognizes free and bound ceramides and its reactivity is species independent. The antibodies against β-actin and p-ERK were obtained from Santa Cruz (Santa Cruz, CA). The antibodies against GCS and GM3 synthase were obtained from NOVUS (Cambridge, UK) and anti-ER-α antibody was from Dako (Carpinteria, CA).

### Cells lines and culture

MCF-7 and MDA-MB-231 human breast cancer cell lines stably transfected with firefly luciferase gene were purchased from Caliper Life Sciences (Hanover, MD). MCF-7 cells were cultured in DMEM medium (Invitrogen, Carlsbad, CA) and MDA-MB-231 cells were grown in DMEM/F-12 (1:1) medium (Invitrogen). Both media were supplemented with 10% fetal bovine serum (Invitrogen) and antibiotic-antimycotic mixture (100 IU/ml penicillin and 100 µg/ml streptomycin; Cellgro). The cells were maintained at 37° C in 5% CO_2_. All experiments were performed when the cells were in the logarithmic phase of growth.

### Luciferase-base bioluminescent optical analysis

The culture cells (10,000 per well) were seeded in flat-bottomed 96-well plate in the proper medium with 10% fetal bovine serum and allowed to grow overnight. The old medium was then replaced with fresh medium containing different concentrations of Sal-B (50, 100, 150, and 200 µM) or doxorubicin (0.1, 0.2, 0.5, and 1 µM). The cells were further cultured for 24 hours and D-luciferin was added to each well and mixed gently (final concentration of 150 µ/ml). The signal was measured with the Xenogen IVIS 200 imaging system (Caliper Life Sciences, Hopkinton, MA, USA). The system is equipped with a highly sensitive, cooled charge-coupled device (CCD) camera and a light-tight specimen box. Imaging and quantification of signals is controlled by the acquisition and analysis software Living Image 3.0. The bioluminescent signal from the cells in each well was expressed as total flux (photons per second [p/s]). At least five replicates were performed in each experiment, and each experiment was repeated at least three times. The representative data were presented.

### Colony formation assay

The colony formation assay was performed as described previously [45]. Briefly, Cells were seeded at a density of 300 cells per well in the 6-well plates, cultured overnight and then treated with Sal-B at different concentrations (1, 10, 20, 50, and 100 µM) for 24 hours. Following treatment, the old medium containing Sal-B was replaced with fresh drug-free medium and the cells were allowed to grow for 10 days to permit colony formation from viable clonogenic cells. Colonies were stained with 0.1% trypan blue in 50% ethanol and were counted manually under microscopy with a grid printed on a transparent plastic sheet to keep track of colonies counted. The colonies containing more than 50 cells were considered to represent a viable clonogenic cell. The experiment was done in triplicate for each treatment.

### Flow cytometry

For cell cycle analysis, the cells from both lines were treated with Sal-B (50 and 100 µM) or doxorubicin (0.1 and 1 µM) for 24 hours and then fixed in chilled 80% ethanol. The fixed cells were incubated in a solution containing 100 µg/mL RNase at 37° C water bath for 45 minutes. Propidium iodide (final concentration of 50 µg/ml) was then added to the cells and incubated in a 37° C water bath for another 15 minutes. To analyze the ceramide level, the fixed cells were sequentially incubated with anti-ceramide primary antibody (1:100) for 2 hours and PE-labeled secondary antibody (1:200) for 30 minutes. The analysis was carried out with a FACStar flow cytometer (Becton Dickinson, San Jose, CA). Ten thousand cells per sample were analyzed, each sample was done in triplicate and the experiment was repeated three times.

### Western blot analysis

Proteins were extracted from the cells with RIPA lysis buffer (Santa Cruz Biotechnology, Santa Cruz, CA) and the protein concentrations were quantified with Bio-Rad protein quantification kit. Whole-cell proteins were separated on 8% SDS-polyacrylamide gel, transferred to the polyvinylidene difluoride membrane (Bio-Rad), and then probed with the indicated primary antibodies overnight at 4° C. Washed blots were then incubated with horseradish peroxidase-conjugated anti-rabbit, anti-mouse, or anti-goat antibody (Santa Cruz Biotechnology), respectively, for one hour at room temperature. Blots were developed and visualized with ECL detection system (Bio-Rad).

### Immunohistochemical staining

Immunohistochemical staining was performed as described previously [45]. Deparaffinized specimens were first labeled with anti-PCNA, Bcl-xL, survivin or ceramide antibody and secondary antibody sequentially. The slides were then stained with streptavidin-horseradish peroxidase and imaged with a microscope equipped with a camera and linked to a computer. For the quantification, ten high-magnification (40×) fields were randomly selected from each slide of tumors and the positive cells were counted. The protein expression was expressed as the average number of positive cells per high magnification field.

### Human tumor xenografts in athymic nude mice

The animal protocol was approved by the Howard University Animal Care and Use Committee. Four-week-old female athymic nude mice (Nu/Nu) were obtained from Harlan Sprague Dawley (Indianapolis, IN). All mice were provided the Harlan Teklad #2018 Global 18% protein rodent diet and water *ad libitum*. Mice were housed in temperature-controlled rooms (74 ± 2° F) with a 12-hour alternating light-dark cycle. MDA-MB-231 cells (3 × 10^6^/50 µL/spot) were subcutaneously injected into the both sides of lower back of mice using a 27-gauge needle. On the day 10 after cell implantation, the mice with tumors (∼5 mm in diameter) were randomly divided into Sal-B treated, doxorubicin treated, and untreated groups (five mice per group), and were administrated Sal-B (80 mg/kg, three times per week), doxorubicin (4 mg/kg, every four weeks) and 0.9% saline (50 µl, three times per week), respectively, by intraperitoneal injection. Mouse weight was measured once a week and tumor size was monitored by manual measurement with a caliper. All mice were sacrificed on day 37.

### Terminal deoxynucleotidyl transferase-mediated dUTP nick end labeling (TUNEL) assay

Apoptotic cell death in tumor xenograft tissue sections was determined by TUNEL assay using the TdT-FragEL DNA Fragmentation Detection Kit (EMD Millipore, Burlington, MA) as described previously [24]. Briefly, sections were digested with proteinase K, and endogenous peroxidase activity was blocked with 3% hydrogen peroxide in 10 mM Tris (pH 8.0). The sections were then placed in equilibration buffer and incubated with TdT enzyme in a humid chamber at 37° C for 1.5 h. The apoptotic nuclei were stained by 3,3′-diaminobenzidine and observed by microscopy. We manually counted the number of positively stained nuclei and the percentage of positive cells versus the total number of cells was calculated.

### Statistical analysis

Values represent the means ± SD of a minimum of three replicate tests. Data were analyzed by the Duncan test following the ANOVA procedure when multiple comparisons were made. Differences were considered significant when *P* < 0.05.

## Abbreviations

Sal-B: Salvinolic acid B
TNBC: triple-negative breast cancer
ER-α: estrogen receptor α
HER2: human epidermal growth factor receptor-2
